# Acetabular Reconstruction Using a Trabecular Metal Cup with a Novel Pelvic Osteotomy Technique for Severe Acetabular Bone Defect

**DOI:** 10.1155/2018/9015727

**Published:** 2018-09-03

**Authors:** Keizo Wada, Tomohiro Goto, Tomoya Takasago, Takahiko Tsutsui, Koichi Sairyo

**Affiliations:** Department of Orthopaedics, Institute of Biomedical Sciences, Tokushima University Graduate School, Tokushima, Japan

## Abstract

**Case:**

A 79-year-old woman with an extreme bone defect after failed cementless total hip arthroplasty underwent revision arthroplasty with a novel technique that involved cutting the anterior iliac bone and sliding it distally to reconstruct the anterior acetabular wall. A three-dimensional printed bone model enabled understanding the details of the bone defect. The clinical outcome at 3 years after surgery was favorable.

**Conclusion:**

The advantages of this technique are twofold, namely, stable fixation of the cup sandwiched between the anterior and posterior walls and reconstruction of the anterior wall using living bone, which allows bone ingrowth into the cup.

## 1. Introduction

Acetabular reconstruction surgery after failed total hip arthroplasty (THA) with severe bone loss is a challenging procedure. Various surgical techniques have been introduced for acetabular reconstruction; however, the outcomes of these techniques are not necessarily reliable [1–3], particularly in the case of severe bone loss or pelvic discontinuity (i.e., a Paprosky 3A or 3B defect) [4]. Stable initial fixation of the implant with precise preoperative planning, assessment of the condition of the residual bone and bone defects, and preservation of as much bone stock as possible are key to achieving a favorable outcome. In this report, we describe the use of a novel technique for pelvic osteotomy assisted by placement of a trabecular metal cup for acetabular reconstruction surgery in a patient with catastrophic bone loss after failed THA. Preoperative planning using a three-dimensional (3D) printed bone model was extremely useful for understanding the relationship between the implant and the host bone.

## 2. Case Presentation

The patient was an elderly woman with osteoarthritis secondary to developmental dysplasia of the right hip who underwent THA using a VerSys® MidCoat Hip System femoral stem and a Trilogy® Acetabular System cup (Zimmer Biomet, Warsaw, IN) at the age of 76 years. Three years later, she developed right groin pain with gradually progressing difficulty in walking. She visited her local hospital and was referred to our hospital for further treatment. Her body weight was 46 kg, and height was 143 cm. She had a medical history of hypertension and asthma, which were controlled by medication. Physical examination revealed a slightly limited range of motion at the right hip (100 degrees of flexion, 0 degrees of extension, 20 degrees of abduction, 10 degrees of adduction, 45 degrees of external rotation, and 10 degrees of internal rotation). Her right leg was approximately 3 cm shorter than the left leg.

**Figure 1:**
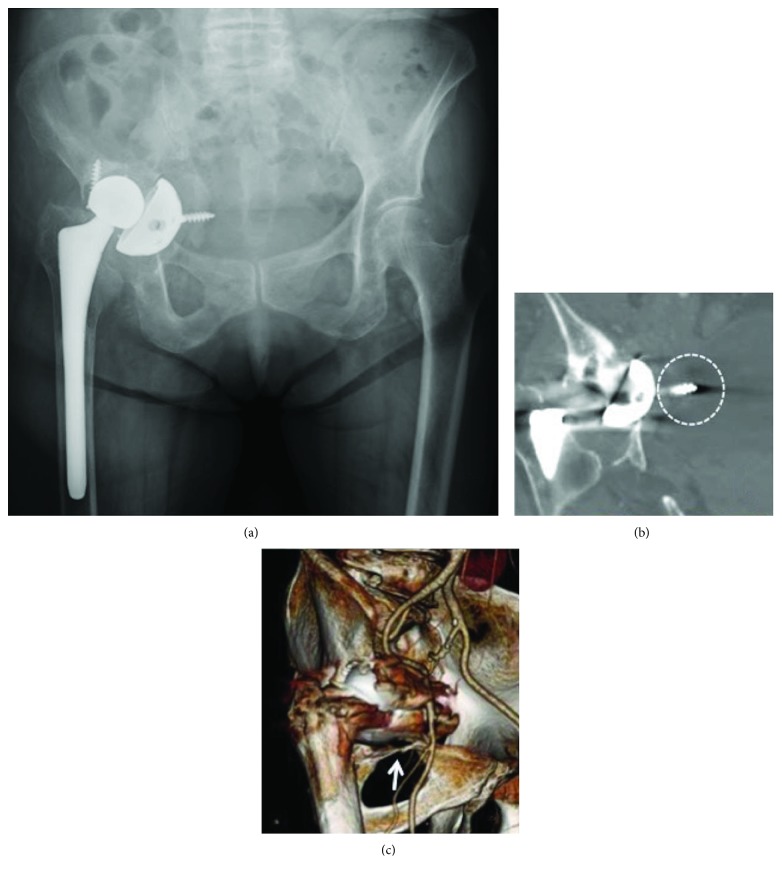
Preoperative plain radiographic (a) and computed tomographic (b, c) images of the pelvis. Coronal computed tomographic image shows a screw pressing the bladder wall (white dotted circle). White arrow indicates a fracture of the superior aspect of the obturator foramen.

**Figure 2:**
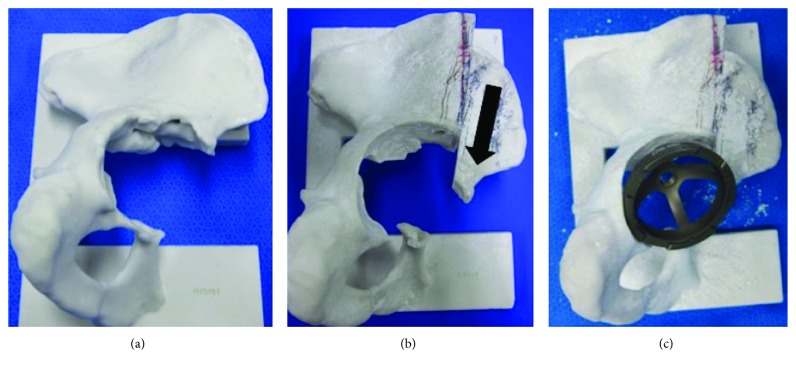
Images from the bone model used for preoperative planning. (a) A bone defect is seen at the acetabulum. (b) We rehearsed the iliac osteotomy and slid the ilium distally (black arrow). (c) The trial cup was placed in the acetabulum.

Anteroposterior pelvic radiography revealed migration of the acetabular cup into the pelvis with destruction of the medial wall of the acetabulum (Figure 1(a)). Computed tomography (CT) showed extensive destruction of bone at the anterior and medial aspects of the acetabulum and a migrated cup screw compressing the bladder wall (Figure 1(b)). The migrated cup was also pushed anteriorly and in contact with the femoral vessels (Figure 1(c)). The remaining bone at the superior aspect of the obturator foramen was fractured, and osteolysis was observed in the posterior column. There were no abnormal findings on the femoral side, and there was no evidence of infection on preoperative culture of joint fluid. A 3D printed bone model was created from a 2 mm slice CT image using Biotec Bones (Zimmer Biomet, Warsaw, IN) to assess the condition of the residual bone at the acetabulum and to allow detailed preoperative planning, including simulation of the iliac osteotomy, acetabular reaming, and implanting of a trial cup to check the relationship between the cup and the host bone (Figure 2). We planned to cut the ilium longitudinally and slide it distally to reconstruct the anterior column of the acetabulum (Figure 2(b)). The acetabulum was gently reamed, and the cup was inserted into the acetabulum, supported by the reconstructed anterior column of the ilium (Figure 2(c)).

**Figure 3:**
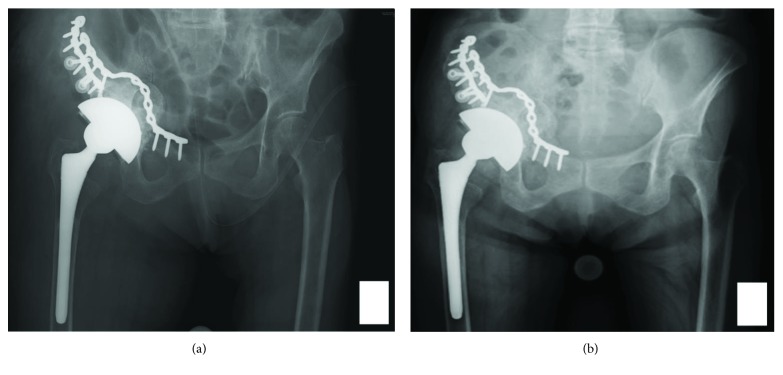
Postoperative radiograph at immediately after surgery (a) and at final follow-up (b).

**Figure 4:**
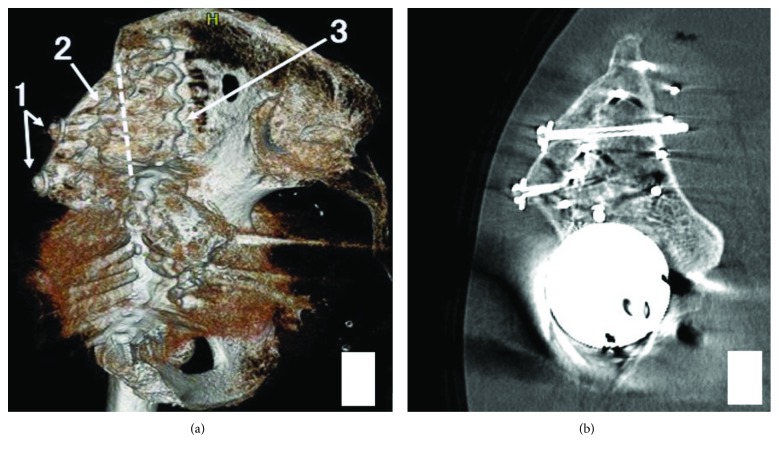
Reconstructed computed tomographic image (a). The dotted line indicates the osteotomy line of the iliac bone. The slid bone is fixed by two screws (1) and a small plate (2), and (3) indicates the reconstruction plate. Cross-sectional computed tomographic image (b).

Reconstruction surgery was performed using a trabecular metal cup (Zimmer Biomet, Warsaw, IN) with pelvic osteotomy and bone grafting using a bridging plate from the pubis to the ilium. First, we removed the migrated cup and screws from inside the pelvis using an ilioinguinal approach in the supine position. Next, we cut the anterior part of the iliac bone and slid it distally and fixed it at the iliac crest with screws and small reconstruction plates (Figures 2(b) and 3(b)). An additional long reconstruction plate bridging the pubis and the iliac bone was inserted to support the medial wall of the acetabulum (Figure 3(b)). The patient was then shifted into the left lateral position for cup placement and bone grafting using a direct lateral approach. A bulk and impacted morselized bone allograft (measuring two and a half times the volume of the femoral head) was used to fill the anterior, medial, and superior aspects of the acetabular bone defect. The acetabulum was then carefully reamed to 68 mm in diameter. Finally, a 68 mm sized trabecular metal cup was placed and fixed to the residual posterior column using 3 screws. The amount of blood loss was 920 ml, and the operation time was 8 hours and 41 minutes. The postoperative course was uneventful. There was no complication around the iliac crest. Partial weight bearing was started from 8 weeks postoperatively, and full weight bearing was allowed at 12 weeks postoperatively. The patient could perform active knee flexion at 2 weeks and straight leg raising at 4 weeks postoperatively. At the most recent follow-up 3 years after surgery, the patient was walking with a T-cane and was independent in activities of daily living with no infection or dislocation of the hip. A plain radiograph at final follow-up showed her limb length discrepancy was 5 mm and revealed no evidence of implant migration (Figure 3). Bone union at the osteotomy site was observed on postoperative plain radiographs and CT images, and there was no evidence of aseptic loosening of the cup (Figure 4).

## 3. Discussion

We were able to achieve an excellent clinical outcome using a novel technique of pelvic osteotomy for acetabular reconstruction surgery in a patient with catastrophic bone loss after failed THA. The advantages of this technique are twofold, that is, stable fixation of the cup sandwiched between the anterior and posterior walls and reconstruction of the anterior wall using living bone, which allow ingrowth of bone into the cementless cup.

There are two main concepts in acetabular reconstruction: one is to achieve rigid containment and recover bone stock using metal mesh or reinforcement devices with bone grafting to stabilize the cemented cup and the other is to achieve bone ingrowth using a cementless cup with or without bone grafting or augmentation devices. Surgical techniques representative of the former concept include a cemented cup with a reinforcement plate [5, 6], a cage [7], or impaction bone grafting [8, 9]. The success of these techniques depends on achieving adequate initial stability followed by a biological response of graft incorporation and remodeling. In a patient with a massive bone defect, a large bone graft is needed, and incorporation or remodeling of the grafted bone takes quite a long time. Therefore, we need to be cautious about the long-term results when using these techniques in patients with severe bone defects [8, 9]. Moreover, when using reinforcement devices, such as the Ganz ring or Kerboull-type acetabular device, the bone at the obturator foramen must be restored for fixation of the distal hook of the device. In our patient, the residual bone at the superior aspect of the obturator foramen was very poor and fractured, so stable fixation could not be achieved using these reinforcement devices.

Trabecular metal component systems (Zimmer Biomet) are reported which may have increased biocompatibility and allow enhanced bone ingrowth and fixation [10]. Currently, a trabecular metal cup with augmentation is probably the most reasonable option for patients with severe bone loss; however, the preoperative 3D printed model revealed that the largest trabecular metal cup available did not extend from the posterior column to the pubis, and the contact area between living bone and the implant would have been too small even if metal augmentation was used. Therefore, we considered that it would be better to reconstruct the anterior wall of the acetabulum using living bone, which could provide anterior support for the implant and allow ingrowth of biological bone in the cup.

The technique described here has several potential risks. The first concern is the possibility of postoperative muscle weakness caused by sliding of the anterior part of the ilium distally, which involves the anterior superior and inferior iliac spines. Theoretically, this could lead to muscle weakness by shortening of the quadriceps and sartorius muscles. However, postoperative muscle recovery was only slightly delayed in our patient; she was able to perform active knee flexion at 2 weeks and active straight leg raising at 4 weeks after surgery and subsequently gained full recovery of muscle strength at the hip. The second concern is that the mechanical strength of the refixed anterior part of the ilium might be weak. We anticipated that even partial bone ingrowth or bone union would be needed to withstand the mechanical stress of weight bearing so did not allow our patient to bear weight until 8 weeks after the surgery. However, a long period of non-weight bearing is generally needed in patients with large bone defects when using the other reconstruction methods. The third issue is that this surgical method was invasive. Therefore, close attention needs to be paid to the patient's general physical status and a comprehensive support strategy that considers all possible complications should be prepared.

We have presented here a novel technique for acetabular reconstruction surgery in patients with massive bone loss secondary to failed THA. Although careful follow-up is needed, we obtained a good short-term outcome. Acetabular reconstruction surgery in patients with catastrophic destruction of the acetabulum is extremely difficult, and there are no standard treatment strategies. Originality and ingenuity in the surgical treatment of individual cases are very important in our quest to improve clinical outcomes.
